# Predicting the co-invasion of two Asteraceae plant genera in post-mining landscapes using satellite remote sensing and airborne LiDAR

**DOI:** 10.1038/s41598-025-16441-3

**Published:** 2025-10-07

**Authors:** Kamil Kędra, Andrzej M. Jagodziński

**Affiliations:** Institute of Dendrology, Polish Academy of Sciences, Kórnik, 62-035 Poland

**Keywords:** *Erigeron* spp., *Solidago* spp., Invasive plants, Natural succession, Novel ecosystems, Machine learning, Ecology, Environmental sciences

## Abstract

The Asteraceae plant family includes the most widespread weedy invaders in Europe, which may jointly inhibit natural succession in degraded land under restoration. The complex local drivers of co-invasions hinder remote sensing (RS) monitoring efforts, as the links between the ecological and the spectral habitat properties are largely unknown. We proposed a comprehensive framework for machine learning modeling of the co-invasion of two *Erigeron* spp. and two *Solidago* spp. in post-mining landscapes of S Poland, using both field data and a combination of Sentinel-2, Landsat 7 and airborne LiDAR RS predictors. Stochastic Gradient Boosting best captured the non-linear dependencies (Accuracy = 0.670–0.886, AUC = 0.675–0.923), and generally outcompeted two other classifiers (Random Forest and Support Vector Machines with a Radial Basis Function Kernel). The field-based functional diversity metrics were the strongest predictors, corroborating improved resistance to invasions by native plant functional richness. In terms of RS data, the most favorable conditions for co-invasion were identified by a narrow range of reflectance in the red-edge interval of a Sentinel-2 image, and constrained by LiDAR-derived vegetation height (for *Erigeron* spp.) and by high land surface temperatures (for *Solidago* spp.). The highest share of patches suitable for co-invasion was consistently found in the low vegetation land cover class, between 36% and 64% cover. We therefore advise considering particular management actions, such as increasing the supply of native seed, thus improving local community resistance to invasions. The proposed methods and openly available RS predictors may facilitate targeted monitoring and cost-effective management interventions.

**Supplementary Information:**

The online version contains supplementary material available at 10.1038/s41598-025-16441-3.

## Introduction

The level of alien plant invasions is particularly high in human-altered, heavily disturbed areas of western and central Europe^1,2^. On the one hand, invasive plants (IPs) benefit from high frequency of ruderal sites and increased propagule pressure in urban and industrial landscapes^3^. On the other hand, high human population densities often coincide with a warm and mild climate, at low altitudes, which are optimal conditions for most IPs^2^. While such broad-scale patterns of plant invasions were addressed by several comprehensive studies^1–4^, there is an urgent need for a finer-scale identification of areas which are highly prone to invasions, to support the remote monitoring efforts^1,5^. Particularly, the phenomenon of coexistence of two or several IPs requires special attention, while the effects of such co-invasions may exceed the effects of any single IP^6–8^.

The Asteraceae plant family has the largest number of alien representatives in Europe^9^, followed by the Poaceae and the Rosaceae families. This is partly due to a very large number and ubiquity of the Asteraceae plants, but also because of the common weedy habit in this plant family^9,10^. Several Asteraceae IPs are at the top of the list of the most widespread alien plant species in Europe^11^, including two species of the *Erigeron* genus (annual or biennial herbs: *Erigeron canadensis* L. and *Erigeron annuus* (L.) Desf.) and two species of the *Solidago* genus (perennial herbs: *Solidago canadensis* L. and *Solidago gigantea* Aiton). These two Asteraceae genera contribute to the highest level of plant invasions in industrial habitats, followed by other human-made habitats, such as arable land, gardens and parks^10,11^. Moreover, *Erigeron* spp. and *Solidago* spp. may co-invade a single vegetation patch, synergistically altering the local environment in a process called invasion meltdown^12,13^.

The field studies on *Erigeron* spp. and *Solidago* spp. co-invasions focused on eastern China, where it is a common phenomenon^6–8,13^. The co-invasion of the North American Asteraceae genera has been reported from a subtropical humid monsoon climate, Anhui Province^6^ and from a similar location in the urban ecosystems in Zhenjiang^7,8^. These studies identified relatively high soil biological activity and organic matter content in co-invaded locations^6,8^, as well as increased overall plant species richness and functional diversity^7^. These findings may sound like positive effects, especially in biologically poor, post-industrial soils; however, few studies have investigated the co-invasion effects of IPs on the diversity of native flora alone, i.e. excluding alien plant species^14^. Moreover, to our knowledge, there are no studies explicitly addressing *Erigeron* spp. and *Solidago* spp. co-invasions in Europe. These Asteraceae representatives were reported from southern Poland, particularly from the post-coal-mining spoil heaps in the Upper Silesia region^15,16^, being local heat islands with maximal summer temperatures reaching and exceeding 50 °C^17^. The *Erigeron* spp. and the *Solidago* spp. were among the most frequent IPs in the degraded landscapes undergoing spontaneous (unassisted) vegetation succession^16,18^.

The remote sensing (RS) data, such as satellite or airborne imagery, offer a great potential for cost-effective filling of the information gaps between the usually sparse field data collection points^5^. The modeling efforts for distribution of Asteraceae IPs have often operated on a large scale (e.g. country-level), using coarse-grained climatic data^19^ or a mixture of climatic, environmental, reflectance and land cover data^1^, based on presence-only IPs records. Lu et al.^19^ assessed the potential distribution of *S. canadensis* in China within a 0.1-degree grid (above 10 km resolution) and identified large areas suitable for future invasion of the species, especially north of the current distribution. More recently, Sittaro et al.^1^ created species distribution models (SDMs; below 10 km resolution) identifying current and future suitable habitats for 46 IPs in Germany (including the *Solidago* spp.). Still, scaling-up of the field-based ecological knowledge using landscape-wide RS proxy variables, through various modeling approaches, may be limited and biased by the unknown, confounding variables working globally in the models^20^. Therefore, both abovementioned studies called for finer-scale assessments, due to the need for revealing hidden constraints, i.e. not detectable using coarse grids^19^, such as local variation in temperatures or because coarse-grained habitat information may fail to explain differences between IPs distributions^1^, while the local habitat properties may be crucial. However, predicting landscape-level IP distributions using fine-scale RS imagery brings several challenges, including: handling large datasets^5^, outcome uncertainty^21^, and probability thresholding^22^. Moreover, models based on the presence-only data may produce biased predictions, with the false-negative (Type II) errors being more frequent than the false-positive (Type I) errors^23^. These issues are increasingly being coped with by implementing machine learning algorithms, due to their higher flexibility over the distributional regression or Bayesian modeling^24^.

**Fig. 1:**
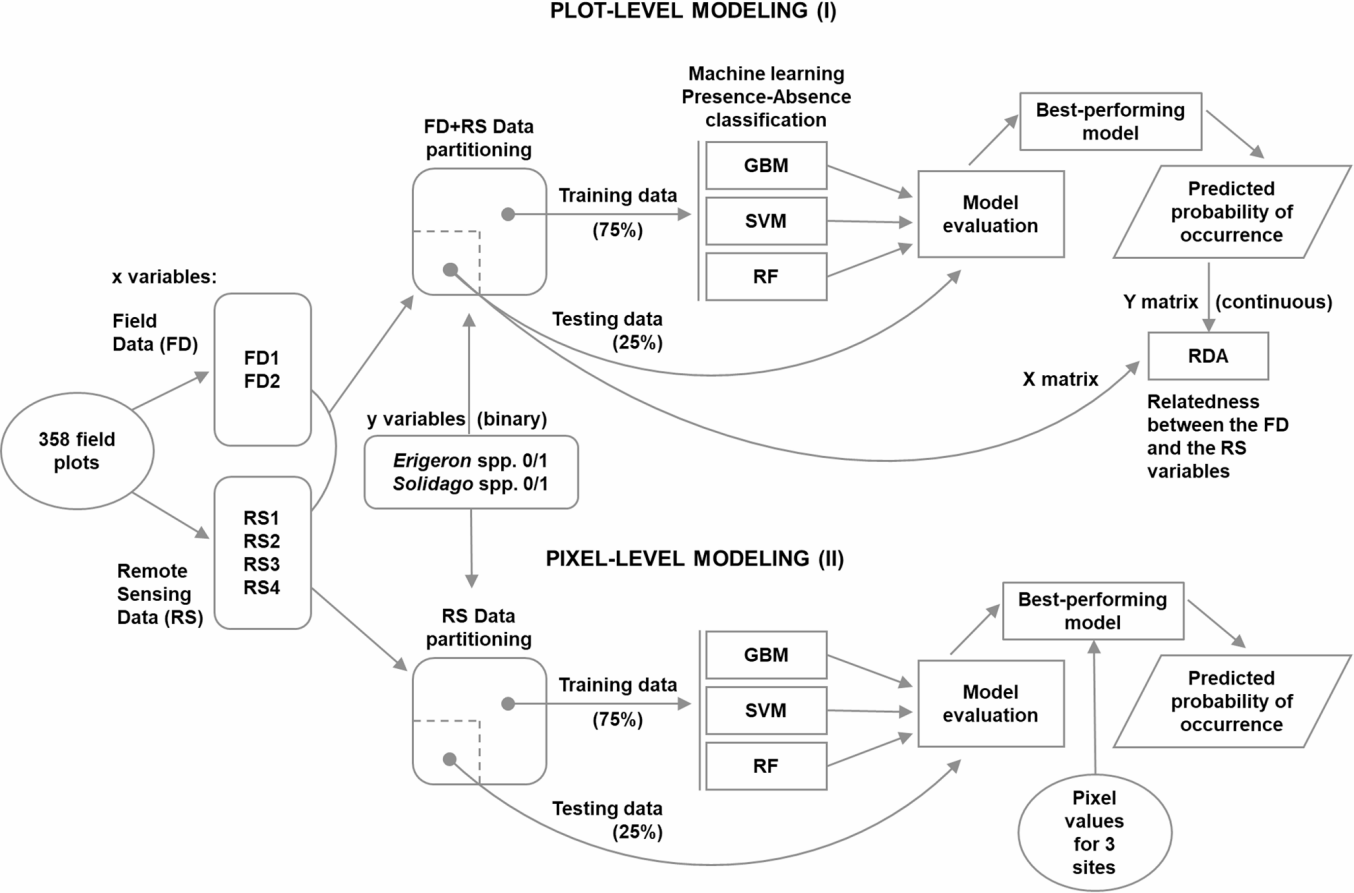
Framework of the two-part modeling procedure, using: (I) combined field data (FD) variables and remote sensing (RS) variables – to uncover the relatedness between the FD and the RS variables using Redundancy Analysis (RDA); (II) RS variables alone – to predict the probability of occurrence of both *Erigeron* spp. and *Solidago* spp. at the pixel- and site-levels; in both (I) and (II), three machine learning techniques are employed and compared: Gradient Boosting Machines (GBM), Support Vector Machines (SVM), and Random Forest (RF).

In this study, we leverage a unique dataset of presence-absence records of *Erigeron* spp. and *Solidago* spp. in post-industrial heterogeneous landscapes of southern Poland, to predict the probability of occurrence of both IP genera alone and jointly (co-invasion). The overarching aim of this study was to propose and evaluate a framework for predicting the co-invasion of the two Asteraceae invasive plant genera using fine-resolution remote sensing data and machine learning methods (Fig. 1). The particular objectives were to:

(i) Compare the predictive power of field data and remote sensing data in modeling *Erigeron* spp. and *Solidago* spp. occurrences;
(ii) Provide an ecologically meaningful interpretation of the remote sensing variables;
(iii) Identify the most favorable conditions for co-invasion of both Asteraceae plant genera, in terms of remotely sensed data and Land Use Land Cover (LULC) types.

The first objective is reached by estimating the relative importance of variables in the IPs presence-absence classification problem, using three different machine learning algorithms. The second objective is addressed by ordinating the IP genera occurrence information using both field data and remote sensing data. Finally, the third objective is achieved by pixel-based predictions of the probability of occurrence for both IP genera over three ecologically distinct sites, and by characterizing the conditions in the overlap areas. We expect that the remotely sensed data may reflect the differences in ecological niches of both IP genera, with *Erigeron* spp. invading under harsher and more initial environmental conditions than *Solidago* spp. Additionally, we hypothesized that the community level invasion of both plant genera is limited by a large number and a high functional richness of native plant species.

## Materials and methods

### Study area and field sampling

**Fig. 2:**
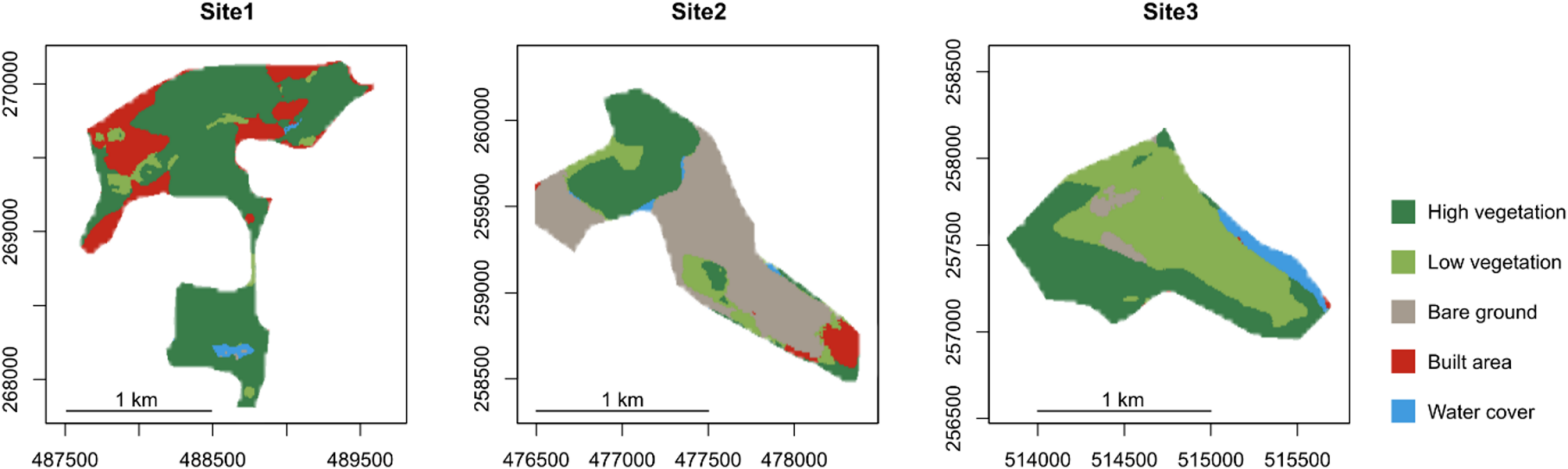
Land cover classes in the three ecologically distinct sites: high share of tree cover (high vegetation) in Site1; high share of bare ground in Site2; high share of low vegetation (jointly: grassland, cropland, shrubland) in Site3; reclassified from the Dynamic World land cover maps^28^, for the year 2022.

The study region is the Upper Silesia in southern Poland, where the coal-mining activities have been transforming the landscape for centuries^25^. The climate is temperate oceanic to continental (from west to east of the region, respectively) with a mean annual temperature ranging from 7 to 9 °C and mean annual precipitation between 700 and 900 mm. The study focuses on the characteristic landscape features of the region: post-mining spoil heaps, which are artificial hills made of mineral (waste) material, containing particles of coal and offering poor biological potential^26^. We selected 28 such heaps, to account for size and successional variation (see Supplementary Fig. S1 for a map). From this number, three sites were further selected as representative of different dominant land cover types (Fig. 2; Table 1), for detailed pixel-based predictions of the IP genera distributions. The field data came from a set of 358 circular plots (28.3 m^2^ each) distributed over the 28 spoil heaps (total area of 1,758.8 ha). At the field plots, all plant species were recorded and vegetation cover by species was estimated in summer 2021^16,27^. The raw field data are available in a public repository (10.6084/m9.figshare.25289401). The *Erigeron* spp. were recorded in 143 plots (40%), the *Solidago* spp. were recorded in 144 plots (40%) and both genera were present in 75 plots (21%).

**Table 1:** Characteristics of the three sites selected for pixel-based predictions of *Erigeron* spp., *Solidago* spp. and co-invasion; class cover percentages were derived from the Dynamic World land cover maps^28^, for the year 2022.

	Area (ha)	Tree cover (%)	Vegetation cover (%)	Bare ground (%)	Built area (%)	Water cover (%)
Site1	167.44	73.4	78.1	0.1	20.5	1.2
Site2	114.39	31.6	41.8	53.0	4.4	0.9
Site3	119.11	41.5	90.8	3.8	0.2	5.2

### Predictor variables datasets

The predictors were grouped under two broad categories: Field Data (FD) and Remote Sensing (RS) data. These categories were further divided into two FD datasets and four RS datasets (Table 2), separating different data sources and types of information: habitat properties, propagule pressure, local climate^3^ or spatial resolution for the RS datasets. Throughout the study we used a projected coordinate reference system with units in meters, for Poland (EPSG 2180).

#### Field data

The first group of the FD variables are plot-level estimates of the following five functional traits: number of species (nbsp), Functional Richness (FRic), Specific Leaf Area (SLA), Seed Mass (SM) and plant Maximal Height (H); all calculated in two variants: for all plant species (“.all” suffix) and for the native plants only (“.nat” suffix). We used the R v.4.4.1^29^ function *FD::dbFD()* in the package “FD” v.1.0.12.3^30^ to derive the plot-level metrics. The input data were plant cover by species measured in the field^27^, and functional traits compiled from several external databases: LEDA^31^, BIEN^32^, BiolFlor^33^, and Pladias^34^. For a small fraction of observations (3% in FRic.all and 9% in FRic.nat) the function *FD::dbFD()* did not reach a convergence and we imputed the missing FRic data using the Random Forest^35^ method (R-squared = 0.72 and 0.73 for FRic.all and FRic.nat, respectively) in the “caret” v.6.0.94 R package^36^, based on the remaining FD1 variables plus leaf dry matter content (the latter not used further in this study). The second FD group includes a single structural trait (Succession), which is the major axis of the nonmetric multidimensional scaling (NMDS) of all plant species (herbs, shrubs and trees) presence-absence data^27^.

#### Remote sensing data

RS1 – two 5-m resolution raster layers recalculated from 1 × 1 m Canopy Height Models (CHMs), representing local average height (CHMmean) and local standard deviation of height (CHMsd). These layers were derived using the *raster::focal()* function in the R package “raster” v.3.6.26^37^. The CHMs are based on an airborne LiDAR (Light Detection and Ranging) scanning campaign, held within the study area in August 2022, using the Riegl VQ780i scanner mounted on an ultralight (KR 030-Topaz) aircraft. The spatial resolution of the LiDAR point clouds was around 20 points m^− 2^.

RS2 – two 30-m resolution raster layers: Normalized Difference Vegetation Index (NDVI) and Land Surface Temperature (LST, in °C), derived from the Landsat 7 ETM + sensor products (atmospherically corrected surface reflectance bands and a thermal band; available at https://developers.google.com/earth-engine/datasets/catalog/LANDSAT_LE07_C02_T1_L2), acquired and processed via the Google Earth Engine web-based facility^38^. NDVI is indicative of habitat properties (greenness, level of physiological stress) and LST is a leading RS variable for local thermal climate determination^39^. Landsat 7 is known for its high radiometric and geometric accuracy, although cloud cover and data gaps are issues that need handling^40^. For NDVI we used the ten highest quality (Tier 1) Landsat 7 images available over the period between the beginning of June and the end of August 2021. The Red band (630–690 nm) and the near infrared (NIR; 770–900 nm) band were pixel-wise merged across the ten images into single bands by the minimal value, thus minimizing the effects of cloud cover and filling any existing data gaps. Subsequently, NDVI was calculated using the formula:

1 $${\text{NDVI = }}\left( {{\text{NIR - Red}}} \right){\text{ / }}\left( {{\text{NIR + Red}}} \right)$$

following^41^. Similarly, for LST the Landsat 7 thermal bands (10400–12500 nm) across the ten 2021 images were pixel-wise merged, but this time by the maximal value (yearly extreme heat), then scaled to Kelvin by using specific constants provided by the data producer^42^, and recalculated to degrees Celsius by subtracting 273.15. We inspected the resulting NDVI and LST images visually (cropped to each spoil heap with a 100-m buffer) for spatial consistency, and while the NDVI layers were of a satisfactory quality, some of the LST images still showed a striped pattern. We therefore extended the range of included Landsat 7 images for this variable by including the two adjacent years (June to August of 2020 and 2022), this resulted in spatially contignous LST images without any data gaps.

RS3 – seven reflectance bands from a Sentinel-2B MSI (MultiSpectral Imager) Level-2A image, covering a wide range of reflectance spectra (between 442 and 1610 nm). The raw, cloud-free Sentinel-2 image (for the 9th of September 2021) was downloaded using the Copernicus Browser^43^ and processed in EnMAP-Box 3 v.3.15^44^ to surface reflectance values. The selected reflectance bands emphasize different habitat properties, such as biomass density (NIR) and moisture content (SWIR1: Short-Wave Infrared)^45^.

RS4 – a group of five spectral diversity indices based on the Sentinel-2 multispectral data. Spectral diversity (local variation in reflectance bands) is thought to explain plant taxonomic and functional diversity^46^, i.e. the spectral variation hypothesis^47^. Such variables are important in the view of the biotic resistance hypothesis^48^, i.e. that species-rich and diverse communities are more resistant to invasions^49,50^. The first two indices (TCDImean and TCDIsd) were calculated using the Tasseled Cap Disturbance Index (TCDI) 10-m resolution maps^51,52^, and inform about the level and variation of local disturbance (within the neighboring and the target pixel), respectively. This information may be indicative of local habitat properties and the magnitude of propagule pressure^3^. The third index is the local minimal value of the Sentinel-2 Leaf area Index (SeLImin)^53^. Low SeLImin values (around zero) indicate presence of bare ground or sparsely vegetated patches (higher invasibility), and high SeLImin values (above 0.5) identify more homogeneous vegetation with high leaf area (lower invasibility). Finally, we included the Rao’s Q (RaoQ) index^54^, which is designed as the remote sensing counterpart of the field-measured Rao’s quadratic entropy^55^. The RaoQ was calculated two-fold, using the standard NDVI map as input, and using the NIR reflectance of terrestrial vegetation:

2 $${\text{NIRv }} = {\text{ NDVI }} \times {\text{ NIR}}$$

which may better represent the radiation absorbed by a canopy, especially for low leaf areas^56^.

**Table 2:** Summary of the variables used in this study: field data (FD1-2) and remote sensing (RS1-4) datasets; the last three columns indicate the assumed environmental significance of the data (see text for the rationale and references); r* = reflectance factor.

Category /Group	No.	Acronym	Source/ resolution	Description	Unit	Habitat properties	Propagule pressure	Local climate
Field Data **FD1**	1,2	nbsp.all nbsp.nat		Number plant species of all (.all) and number of native plant species (.nat)	-	×	×	
	3,4	FRic.all FRic.nat		Functional Richness for all plant species (.all) and only for the native plants (.nat)	-	×		
	5,6	SLA.all SLA.nat		Community Weighted Mean Specific Leaf Area for all plant species (.all) and only for the native plants (.nat)	cm^2^ g^− 1^	×		×
	7,8	SM.all SM.nat	n.a.	Community Weighted Mean Seed Mass for all plant species (.all) and only for the native plants (.nat)	g		×	
	9,10	H.all H.nat		Community Weighted Mean Maximal Height for all plant species (.all) and only for the native plants (.nat)	m	×		
**FD2**	11	Succession		Major axis of the nonmetric multidimensional scaling (NMDS) of all plant species presence-absence data ^27^	-	×	×	×
Remote Sensing data **RS1**	1	CHMmean	LiDAR 5 m	Average value in a 5 × 5 cell window of a 1-m resolution LiDAR Canopy Height Model	m	×		×
	2	CHMsd		Standard deviation value in a 5 × 5 cell window of a 1-m resolution LiDAR Canopy Height Model	m	×	×	
**RS2**	3	NDVI	Landsat 7 30 m	Normalized Difference Vegetation Index	-	×		×
	4	LST		Land Surface Temperature	°C			×
**RS3**	5	Aerosols	Sentinel-2 60 m	Aerosols band (442 nm)	r*		×	×
	6	Blue	Sentinel-2 10 m	Blue band (492 nm): soil and vegetation discrimination; chlorophyll and carotenoids absorption	r*	×		
	7	Green		Green band (559 nm): strongly reflected by green foliage	r*	×		
	8	Red		Red band (665 nm): strongly reflected by stressed and dead foliage; chlorophyll absorption	r*	×		
	9	RedEdge1	Sentinel-2 20 m	Red Edge band (704 nm): differentiates between vegetation types	r*	×		
	10	NIR	Sentinel-2 10 m	Near InfraRed band (833 nm): biomass content	r*	×		
	11	SWIR1	Sentinel-2 20 m	Short-Wave Infrared (1610 nm): moisture content of soil and vegetation	r*	×		
**RS4**	12	TCDImean	Sentinel-2 10 m	Focal average value of the Tasseled Cap Disturbance Index in a 5 × 5 cell moving window	-	×	×	
	13	TCDIsd		Focal standard deviation value of the Tasseled Cap Disturbance Index in a 5 × 5 cell moving window	-	×	×	
	14	SeLImin		Focal minimal value of the Sentinel-2 leaf area Index in a 5 × 5 cell moving window	-	×	×	×
	15	RaoQ_NDVI		Rao’s quadratic entropy index in a NDVI layer 5 × 5 cell moving window	-	×		
	16	RaoQ_NIRv		Rao’s quadratic entropy index in a NIRv layer 5 × 5 cell moving window	-	×		

### Statistical analyses

#### Workflow overview

The modeling framework was divided into two parts (Fig. 1): in the first part we used a fused dataset of the field (FD1-2) and the remote sensing (RS1-4) datasets as predictors of *Erigeron* spp. and *Solidago* spp. presence-absence, to compare the predictive power of both FD and RS datasets, and to establish relatedness between them (our first two objectives). In the second part we used solely the RS variables as predictors; although these data are available for every pixel of all the included sites, the spatial resolution differed (between 5 and 60 m, see Table 2 for a detailed list). The goal of the second part is to create site-level maps of the IPs genera and co-invasion and to evaluate and compare the most favorable conditions in all cases (the third objective of the study). We expect that the finest resolution data (LiDAR CHM-derivatives) will play a major role in the models and thus the final resolution of both the Asteraceae genera distribution maps and the co-invasion map will vary considerably at the 5-m resolution. In both modeling parts we largely relied on the “caret” v.6.0.94 R package^36,57^, providing functions for training and evaluating different classification machine learning algorithms. The application or combination of several such statistical techniques is often recommended to improve the prediction quality^1^ and we chose three such methods: Stochastic Gradient Boosting (GBM), Support Vector Machines (SVM) and Random Forest (RF). These algorithms are described below in more detail, in a separate Section.

In both modeling parts, we first created a data partition into training and testing datasets (75% and 25% of observations, respectively) using the function *caret::createDataPartition()*, to balance the class distributions within the splits. The models were trained using the function *caret::train()*, implementing a ten-fold cross-validation, repeated ten times. The criterium for tuning hyperparameters was the Area Under the receiver operating characteristic Curve (AUC). We identified and compared individual variable importance in each modeling approach using the *caret::varImp()* function, and we evaluated the partial effects of the most important predictors using the *pdp::partial()* function in the R package v.0.8.2 “pdp”^58^. Finally, the testing data were used to compare model performance between the different machine learning techniques. We used the following classification performance metrics:

3 $${\text{Sensitivity = TP/}}\left( {{\text{TP + FN}}} \right)$$

4 $${\text{Specificity }} = {\text{ TN}}/\left( {{\text{TN }} + {\text{ FP}}} \right)$$

5 $${\text{Precision }} = {\text{ TP}}/\left( {{\text{TP }} + {\text{ FP}}} \right)$$

6 $${\text{F1}} = {\text{ 2}}\times {\text{ }}\left( {{\text{Precision }} \times {\text{ Sensitivity}}} \right)/\left( {{\text{Precision }} + {\text{ Sensitivity}}} \right)$$

7 $${\text{Accuracy }} = {\text{ }}\left( {{\text{TP }} + {\text{ TN}}} \right)/\left( {{\text{TP }} + {\text{ FP }} + {\text{ TN }} + {\text{ FN}}} \right)$$

where TP, FP, TN, FN are the numbers of: true positive, false positive, true negative and false negative cases, respectively. We also accounted for model uncertainty by including standard deviation (SD) of AUC from the internal cross-validation^21^.

#### Machine learning algorithms

In this study we implemented and compared three machine learning algorithms (Table 3): RF^35^, GBM^59^ and SVM^60^, that have been used in similar modeling tasks^1^. Stochastic Gradient Boosting and Random Forest are both based on ensembles of decision trees^35,59^; however, RF and GBM differ in the model training procedures. Random Forest uses the bagging technique (independent learning of individual trees) and GBM implements the boosting technique (sequential learning of decision trees)^35,59^. These result in differences between model performances: RF is often reported as more robust to outliers and overfitting, while GBM may be more accurate, but somewhat prone to noise in the data and requires more parameters for tuning than RF (Table 3)^61^. In the case of RF, only a single hyperparameter was tuned: mtry (number of variables randomly sampled as candidates at each split); in GBM there were four hyperparameters: n.trees (total number of trees to fit), interaction.depth (the maximum depth of each tree, e.g. defining an additive model or a model with up to n-way interactions), shrinkage (the learning rate or step-size reduction parameter), n.minobsinnode (the minimum number of observations in the terminal nodes of the trees); shrinkage and n.minobsinnode were kept constant (at 0.1 and 20, respectively). In contrast to RF and GBM, Support Vector Machines is a kernel-based machine learning method, which maps the input data into a high dimensional feature space and maximizes the width of the margin between classes^62^. We used the SVM with Radial Basis Function Kernel, to allow a nonlinear class boundary. SVM had an intermediate number of hyperparameters to tune: C (Cost) and sigma (Radial Basis Function sigma).

**Table 3:** Overview of the three machine learning algorithms used in this study, including the implementation in R programing (Libraries) and a list of model hyperparameters (Tuning Parameters).

Model	Acronym	Libraries (citation)	Tuning Parameters
1. Stochastic Gradient Boosting	GBM	gbm v.2.2.2 ^63^, plyr v.1.8.9 ^64^	n.trees, interaction.depth, shrinkage, n.minobsinnode
2. Support Vector Machines with Radial Basis Function Kernel	SVM	kernlab v.0.9.32 ^62^	sigma, C
3. Random Forest	RF	randomForest v.4.7.1.1 ^65^	mtry

#### Multivariate ordination

Redundancy Analysis (RDA) is an appropriate direct canonical analysis for ordination of species field data (Y matrix), such as species cover, composition etc., under the constraints of a set of environmental variables (X matrix)^66^. It is also valid for presence-absence data, but requires a transformation of the Y matrix to reduce the number of zeros and to avoid the double zeros problem^67^. In this work we propose another workaround for this problem, i.e. we used the testing dataset (*n* = 88) to predict the probability of occurrence (continuous output) of the two Asteraceae IP genera, using the best performing model described in the previous Section. Consequently, we used a corresponding dataset of the FD and RS explanatory data. Next, using such prepared X and Y matrices we constructed the RDA model using the function *vegan::rda()* in the R package “vegan” v.2.6.6.1^68^. To assess the relatedness between the different components of the X matrix (FD and RS) we applied a standard ordination plot method (type 2 scaling: the effects of explanatory variables). We also analyzed the Variance Inflation Factors (VIF), which may identify redundant explanatory variables (VIF > 10), and compared the extracted RDA scores (biplot coordinates of the arrows representing variables).

#### Pixel-based site-level predictions

Following the procedure of model evaluation and selection of the best-performing machine learning method (Fig. 1) we fitted the model of the selected type using a full set of observations (n = 358). This model was again trained using the *caret::train()* function^57^ with a ten-fold cross-validation, repeated 10 times. We extracted the cross-validated Sensitivity, Specificity and AUC of the final tuned models (for *Erigeron* spp. and for *Solidago* spp.). For the next step, we extracted the central coordinates (landmarks) for each pixel of the finest-resolution RS dataset (LiDAR CHM, 5-m resolution), for each of the three model sites (Fig. 2, Table 1). We kept all raster layers in their original resolutions, but the images were projected to a common coordinate reference system (CRS EPSG 2180), we then extracted the landmark values of all coarser-resolution layers using the *raster::extract()* function in the “raster”^37^ R package. Subsequently, the predicted probabilities of occurrence of both Asteraceae genera were obtained using the *stats::predict(type=“prob”)* function. The outcomes were analyzed in the form of probability gradients (from 0 to 1) and in a binarized form. While it is known that such binarization is not straightforward and probability threshold depends on target prevalence and model performance^69^, we applied the method that minimizes the difference between Sensitivity and Specificity^22^. To do this, we extracted the cross-validation predictions for each genus and binarized them using a sequence of 100 candidate threshold values (between 0.01 and 1). We then calculated the differences between Sensitivity and Specificity (assuming co-invaded plots as TP) and identified the threshold associated with the lowest difference for each cross-validation data set. The mean threshold values (0.457 for *Erigeron* spp. and 0.387 for *Solidago* spp.) were used to create the binary co-invasion layers, with positive class indicating a plausible probability of occurrence of both Asteraceae genera in the three selected sites. The statistical analysis included comparison of the absolute (in hectares) and relative (percentage) predicted cover of *Erigeron* spp., *Solidago* spp. and of the co-invaded patches; as well as in the reclassified land use land cover classes^28^, available at a 10-m resolution. We applied four broad land cover classes: high vegetation (high probability of mature trees, but also dense high shrubs), low vegetation (grasslands, croplands and shrublands), bare ground (low probability of any vegetation) and built area (high proportion of roads and impervious surfaces). The site-specific averages of the most important RS predictors were compared using the Welch two-sample t-test (0.05 significance level) and Cohen’s d, to identify the effect size^70^. We used standard R histogram plotting to visualize the differences in remotely sensed niches for *Erigeron* spp., *Solidago* spp. and co-invasion.

## Results

### Combined field and remote sensing data

**Fig. 3:**
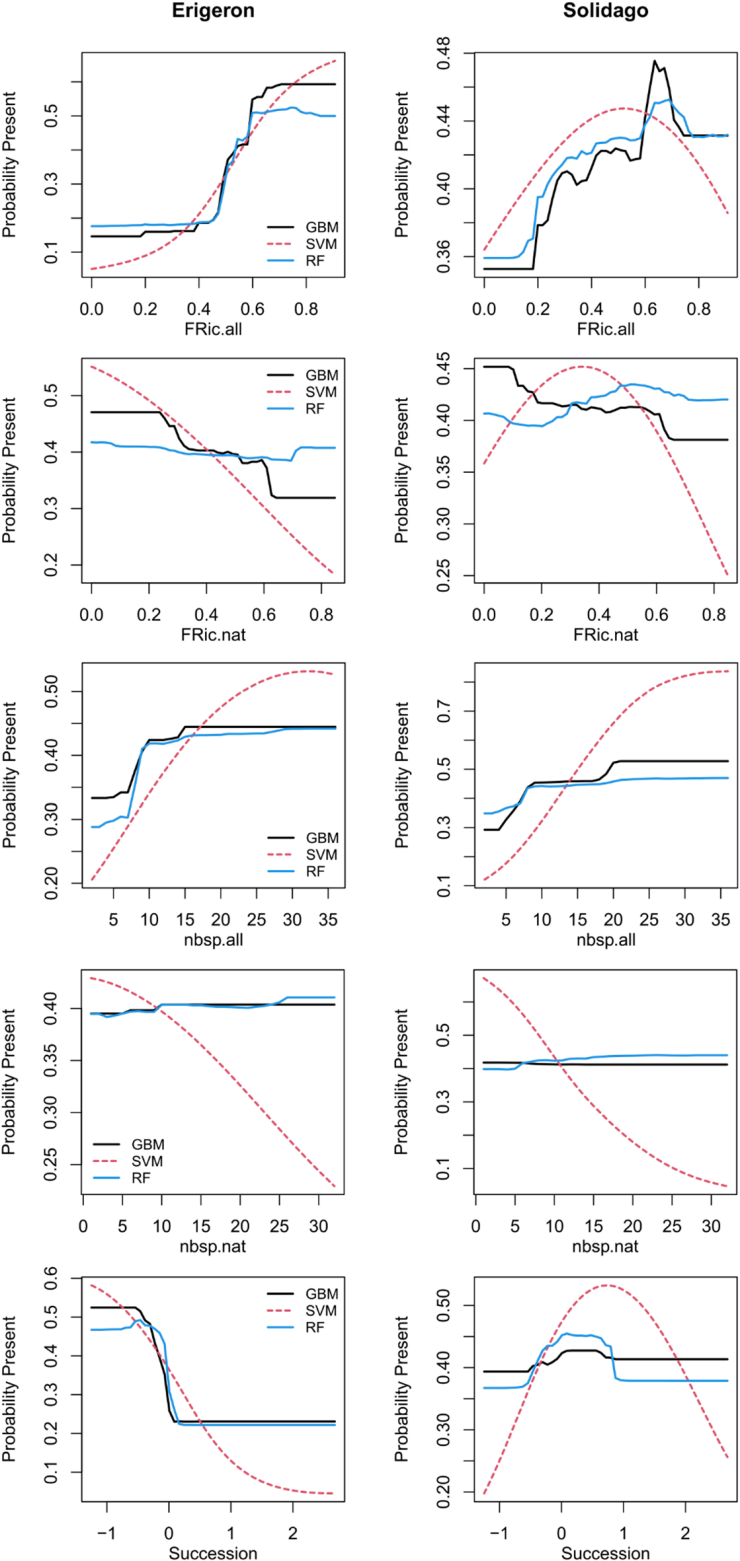
Partial dependence plots in the probability fraction mode, based on *Erigeron* spp. or *Solidago* spp. presence-absence classifications, for five field data variables: Functional Richness (FRich) and number of species (nbsp) for all plant species (.all) and for native species only (.nat), and successional gradient (Succession); different line colors denote different machine learning algorithms (GBM – black solid, SVM – red dashed, RF – blue solid).

Classifier performance differed by the target genus (Table 4). It was generally better for the *Erigeron* spp., with RF (F1 = 0.88, Accuracy = 0.91) slightly outcompeting GBM (F1 = 0.85, Accuracy = 0.87) and considerably outcompeting SVM (F1 = 0.81, Accuracy = 0.85). All three machine learning techniques attained poorer predictions of *Solidago* spp. occurrence, with GBM (F1 = 0.69, Accuracy = 0.78) performing better than SVM (F1 = 0.64, Accuracy = 0.76) and RF (F1 = 0.61, Accuracy = 0.74). Similarly, model uncertainties were higher for *Solidago* spp. (SD of AUC between 0.079 and 0.083) than for *Erigeron* spp. (between 0.050 and 0.054). In terms of the FD predictor variables importance, there was a good agreement across the modeling methods and target genera (Supplementary Tables S1, S2). The overall Functional Richness (FRic.all) and number of species (nbsp.all), as well as the successional gradient (Succession) were the most relevant variables. Functional Richness for native species only (FRic.nat) was close behind the aforementioned predictors, in models for both genera. Importantly for our first objective, the RS data variables had generally lower importance than those top FD variables, and the most relevant RS predictors differed by the target genus. For the *Erigeron* spp. models, LST (representing local thermal climate) was the most important RS variable, followed by TCDImean (local disturbance) and CHMmean (fine-scale vegetation height). For the *Solidago* spp. models, Aerosols (Sentinel-2 first band) was the most relevant, followed by SeLImean and NDVI (both representing habitat properties and vegetation greenness), but LST was also high in the ranking. The partial dependence plots for the most influential FD variables revealed increasing probability of both Asteraceae genera occurrence with increasing FRic.all and nbsp.all, but the probabilities decreased with increasing FRic.nat in the GBM and SVM models (Fig. 3). The optimal values of Succession were low for *Erigeron* spp. and intermediate for *Solidago* spp.

**Table 4:** Evaluation metrics for machine learning *Erigeron* spp. and *Solidago* spp. presence-absence classification models (GBM, SVM, RF) fitted with fused field and remote sensing data; the highest values for each genus are in bold (not tested for differences).

	*Erigeron*			*Solidago*		
	GBM	SVM	RF	GBM	SVM	RF
AUC (± SD)	**0.923** (± 0.054)	0.921 (± 0.051)	0.920 (± 0.050)	0.768 (± 0.083)	**0.795** (± 0.079)	0.782 (± 0.082)
Sensitivity	0.829	0.771	**0.857**	**0.656**	0.594	0.563
Specificity	0.925	0.906	**0.943**	**0.857**	**0.857**	0.839
Precision	0.879	0.844	**0.909**	**0.724**	0.704	0.667
F1	0.853	0.806	**0.882**	**0.689**	0.644	0.610
Accuracy	0.886	0.852	**0.909**	**0.784**	0.761	0.739

### Relatedness between field and remote sensing data

**Fig. 4:**
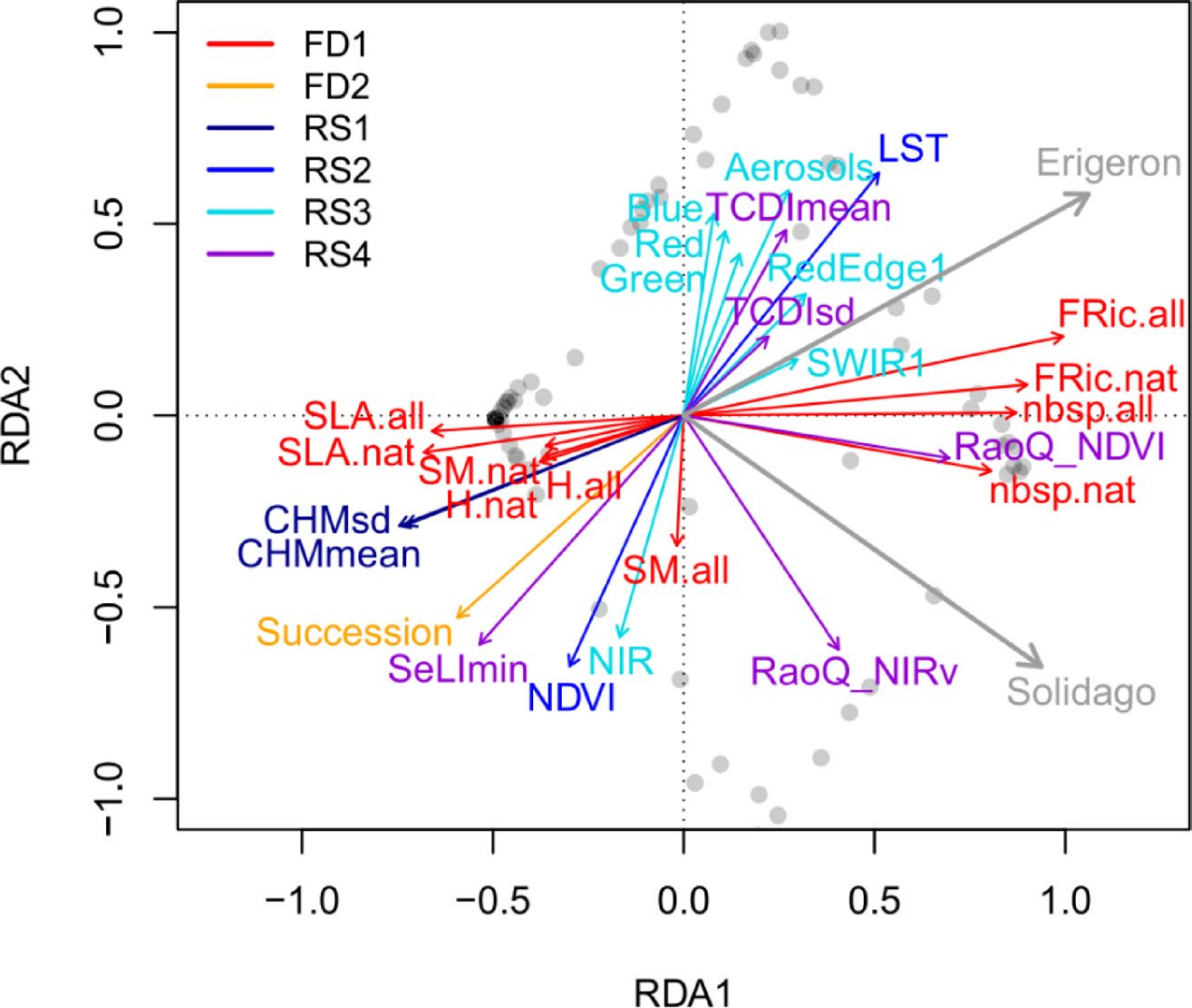
Redundancy Analysis (RDA) ordination of *Erigeron* spp. and *Solidago* spp. probability of occurrence (gray arrows) using both Field Data (FD) and Remote Sensing data (RS); explanatory sub-datasets (FD1-2, RS1-4) are highlighted by different colors (see “Category/Group” in Table 2 for definitions).

Most of the FD and RS variables contributed to the major RDA axis, while the differences between the *Erigeron* spp. and the *Solidago* spp. were better represented by the second RDA axis (Fig. 4 and Supplementary Table S3). In the first RDA axis, the field-based taxonomic and functional diversity metrics and the RS Rao’s entropy (RaoQ_NDVI) were opposed to the community weighted functional traits and RS LiDAR-based height metrics (CHMmean, CHMsd). This result is relevant for our second objective on establishing the linkages between the traditional field-based ecological measures and the RS metrics. The second RDA axis was dominated by the RS variables, with LST, most of the Sentinel-2 bands and local disturbance metrics (TCDImean, TCDIsd) being opposed to NDVI, NIR, SeLImin and field-based Succession. The former subset was positively related to the *Erigeron* spp. probability of occurrence and the latter subset was positively related to the *Solidago* spp. probability of occurrence. The Variance Inflation Factors were generally lower in the FD datasets (Supplementary Table S3a). The lowest VIF values (indicating distinct variables, with little collinearities) in FD were in the cases of Seed Mass and Specific Leaf Area (between 1.55 and 5.21). The highest VIF values were for the three optical Sentinel-2 bands (Red, Green and Blue; up to 513.94) and for TCDImean (296.11). There were only two RS variables with low VIF (below 10): TCDIsd and CHMsd, both representing local variation in habitat properties.

### Effects of remote sensing variables

**Fig. 5:**
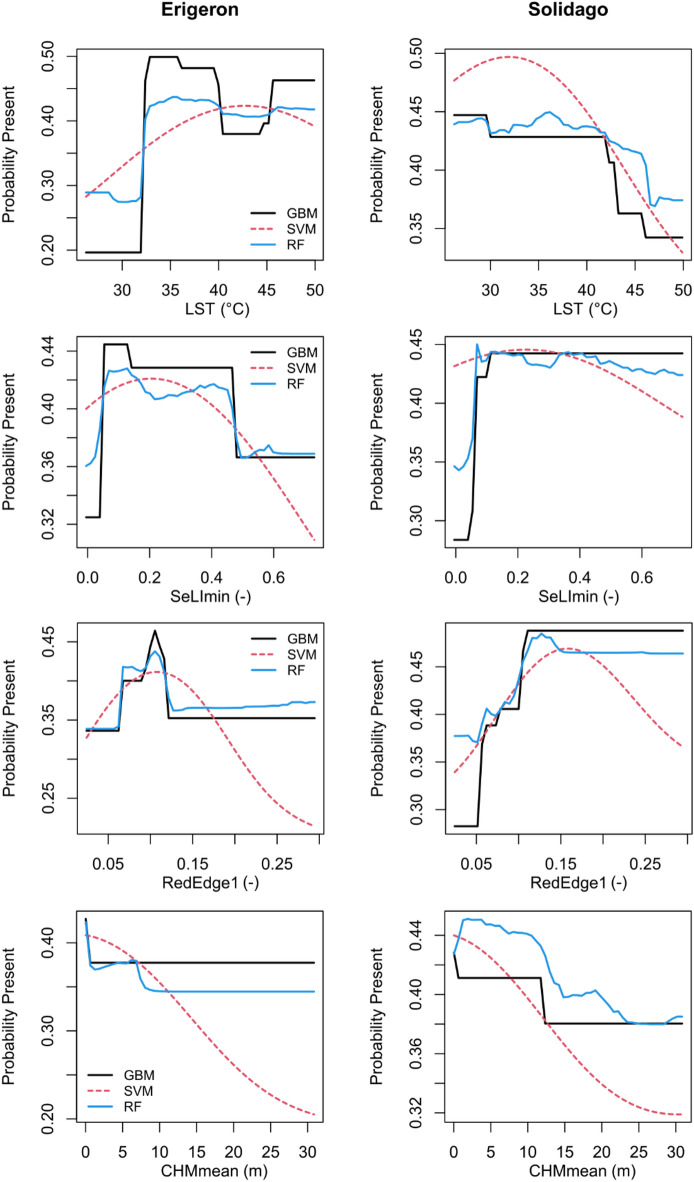
Partial dependence plots in the probability fraction mode, based on *Erigeron* spp. or *Solidago* spp. presence-absence classifications, for four remote sensing variables: LST, SeLImin, RedEdge1 and CHMmean; different line colors denote different machine learning algorithms (GBM – black solid, SVM – red dashed, RF – blue solid).

Removing the FD variables from all models decreased all model performances (Table 5). The largest drop was for the *Erigeron* spp. RF. Generally, the *Erigeron* spp. models suffered more from neglecting the FD data than the *Solidago* spp. models, which resulted in more similar model performances across the target genera. Still, the *Erigeron* spp. classifiers performed better, with the GBM (F1 = 0.67, Accuracy = 0.72) outcompeting both RF (F1 = 0.61, Accuracy = 0.70) and SVM (F1 = 0.58, Accuracy = 0.67). In the case of *Solidago* spp., RF (F1 = 0.53, Accuracy = 0.69) performed slightly better than GBM (F1 = 0.52, Accuracy = 0.67) and SVM (F1 = 0.51, Accuracy = 0.67). Model uncertainties were slightly higher for *Solidago* spp. (SD of AUC between 0.089 and 0.096) than for *Erigeron* spp. (between 0.076 and 0.084). Importantly, for the *Solidago* spp. the GBM was the only classification model where none of the performance metrics dropped below 0.5 (which was the GBM Sensitivity score or true positive rate). In all cases, Specificity (true negative rate; here between 0.72 and 0.82) was higher than Sensitivity, and considering both metrics GBM was the best performing modeling approach. Land Surface Temperature, TCDImean and CHMmean attained high importance in the *Erigeron* spp. classifiers (Supplementary Table S4) after removal of the FD predictors; additionally, SeLImin and RedEdge1 advanced in importance (two variables related to habitat properties and succession). Similarly, in the case of *Solidago* spp. classifiers, Aerosols, SeLImin and NDVI maintained high importance (Supplementary Table S5) compared to the full-data models. Moreover, SWIR1 increased in importance, particularly in the GBM. The partial dependence analysis for the most influential RS predictors revealed some clear differences between *Erigeron* spp. and *Solidago* spp. (Fig. 5). In *Erigeron* spp. models, increasing LST was related to a sharp increase in the probability of occurrence, particularly in the LST range between 30 and 35 °C. In contrast, the highest probability of *Solidago* spp. occurrence was under low or intermediate LST (depending on the modeling approach). The GBM and RF classifiers identified a narrow optimum in RedEdge1 for the *Erigeron* spp. (around 0.1), while in *Solidago* spp. the probability of occurrence increased with increasing RedEdge1, up to around 0.15, and then plateaued. The effects of fine-scale vegetation height (CHMmean) were also distinctly different in the two Asteraceae genera: the *Erigeron* spp. probability of occurrence decreased steeply within the height range up to 5 m, and the probability of Solidago spp. occurrence decreased much slower, plateauing at CHMmean between 10 and 25 m (depending on the machine learning method).

**Table 5:** Evaluation metrics for machine learning *Erigeron* spp. and *Solidago* spp. presence-absence classification models (GBM, SVM, RF) fitted with remote sensing data; the highest values for each genus are in bold (not tested for differences).

	*Erigeron*			*Solidago*		
	**GBM**	**SVM**	**RF**	**GBM**	**SVM**	**RF**
AUC (± SD)	0.803 (± 0.084)	**0.807** (± 0.079)	0.805 (± 0.076)	0.675 (± 0.096)	0.680 (± 0.094)	**0.690** (± 0.089)
Sensitivity	**0.714**	0.571	0.571	**0.500**	0.469	0.469
Specificity	0.717	0.736	**0.792**	0.768	0.786	**0.821**
Precision	0.625	0.588	**0.645**	0.552	0.556	**0.600**
F1	**0.667**	0.580	0.606	0.525	0.508	**0.526**
Accuracy	**0.716**	0.670	0.705	0.670	0.670	**0.693**

### Predicted co-invasion

**Fig. 6:**
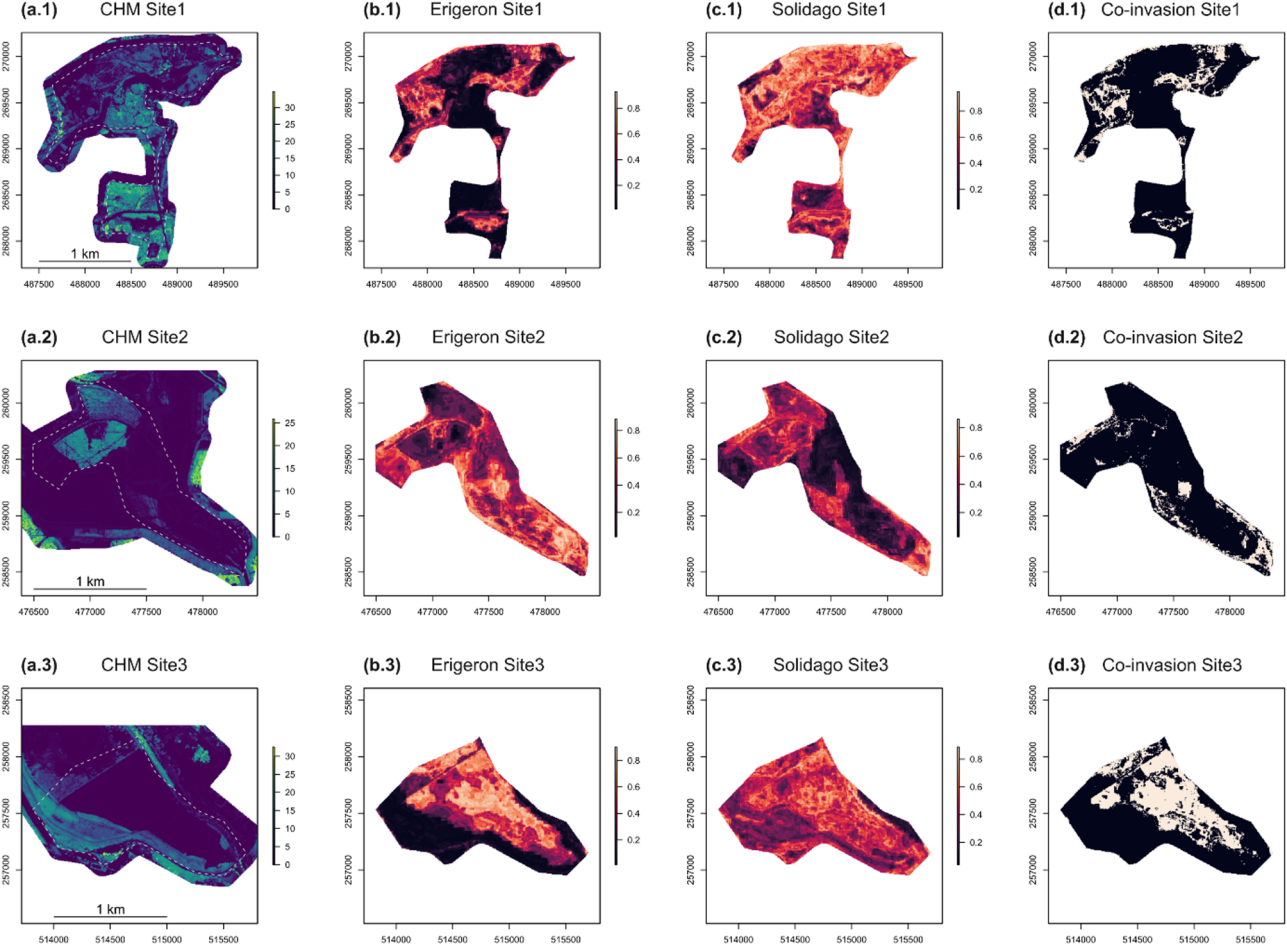
Site level (Site1-3) Canopy Height Models (CHMs, in meters; a.1–3) and GBM-predicted probabilities of *Erigeron* spp. occurrence (b.1–3), *Solidago* spp. occurrence (c.1–3) and both genera co-occurrence (Co-invasion; d.1–3); (b.1–3) and (c.1–3) are probability gradient maps, while (d.1–3) are binarized maps for both the Asteraceae genera; in b-d, dark colors denote low or zero probability of occurrence and bright colors indicate high probability of occurrence.

**Fig. 7:**
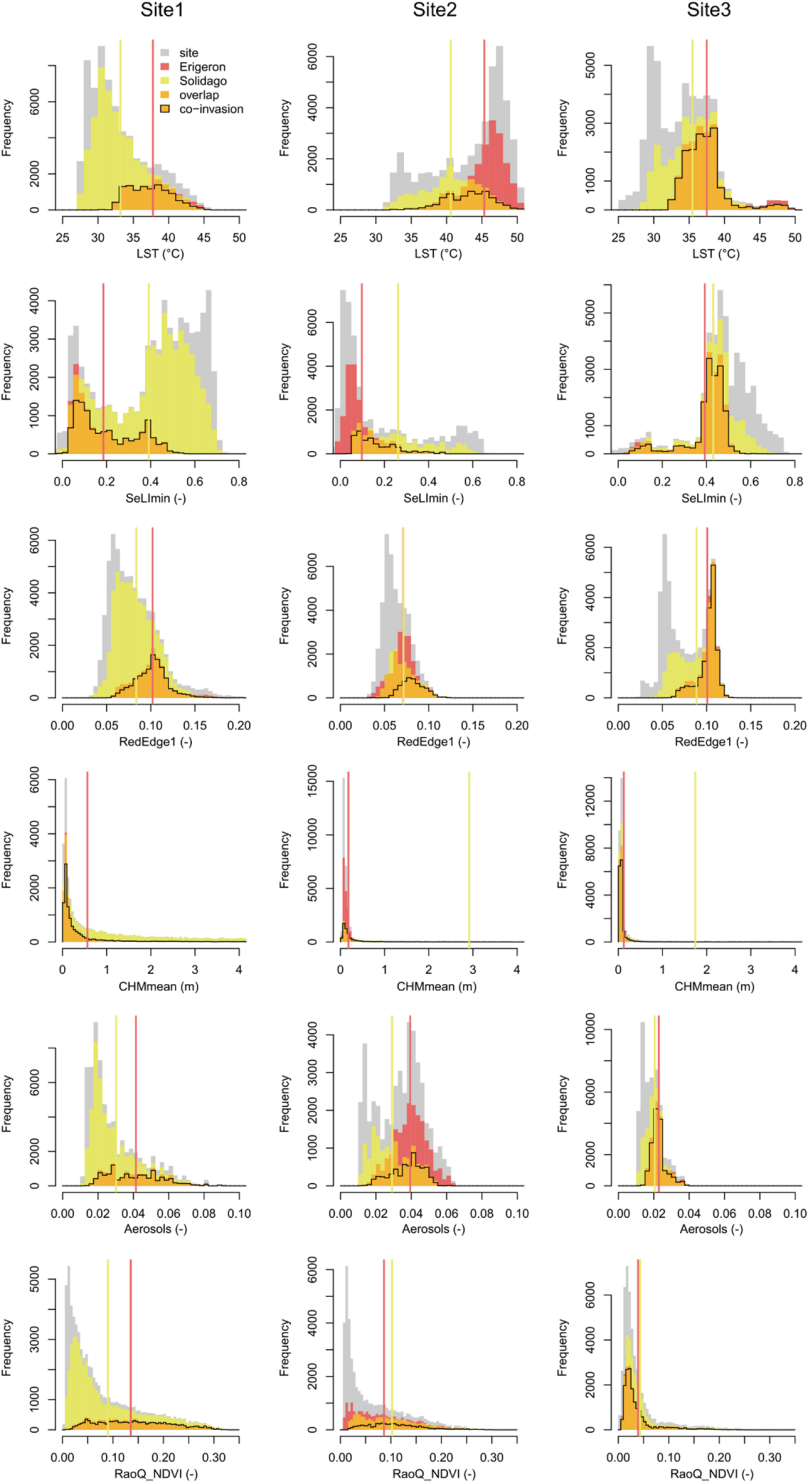
Site-level distributions of selected remote sensing variables pixel values (Site1-3, gray fill) and subsets identified by the GBM predictions for *Erigeron* spp. occurrence (Erigeron, red fill), *Solidago* spp. (Solidago, yellow fill) occurrence or both genera co-occurrence (Co-invasion, orange fill with black outline); vertical lines indicate group means.

**Fig. 8:**
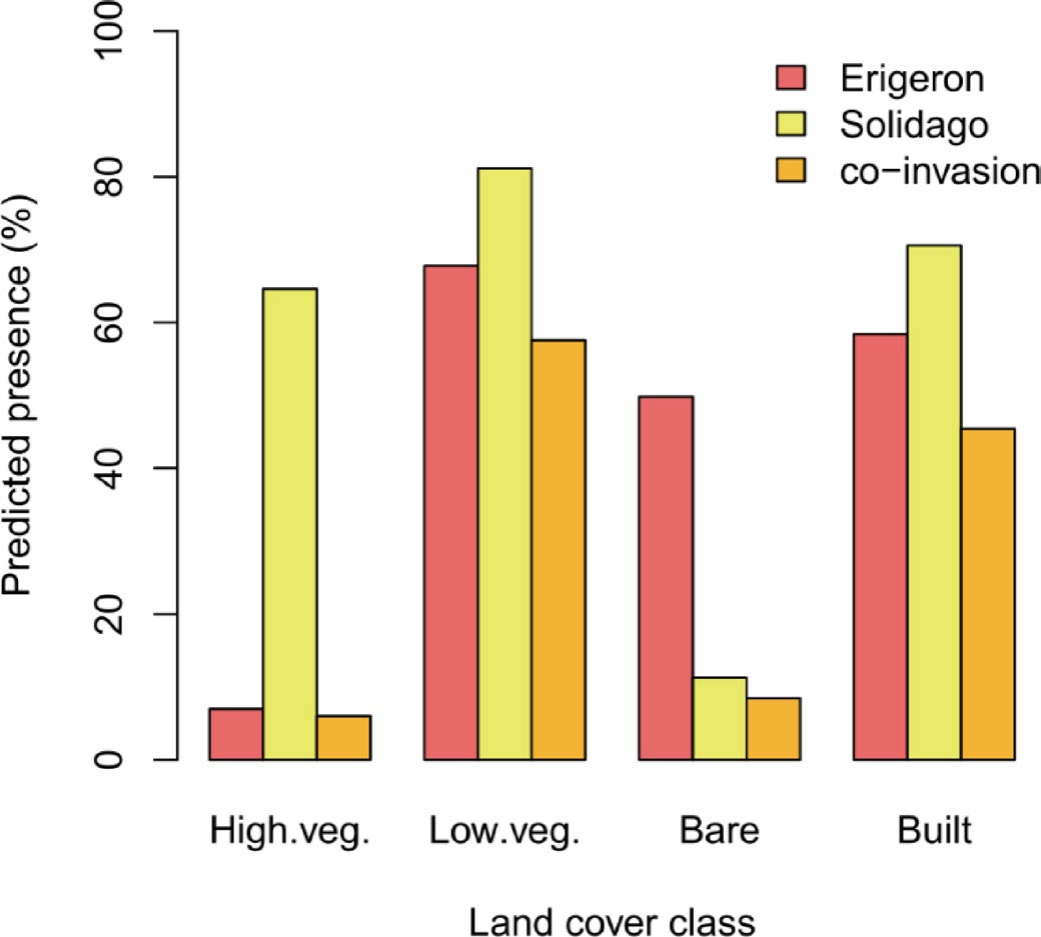
Combined percentage predicted presence across the three selected sites, for *Erigeron* spp., *Solidago* spp. and both genera co-occurrence (co-invasion) in four broad land cover classes (reclassified from Dynamic World LULC^28^: high vegetation (High.veg.), low vegetation (Low.veg.), bare ground (Bare) and built area (Built); see Table 9 for exact values.

Stochastic Gradient Boosting was chosen for site-level predictions (Tables 4 and 5; see Supplementary Table S6 for tuned hyperparameter values). The final GBM models (fitted with all available observations, *n* = 358) again attained mostly higher performance statistics for the *Erigeron* spp. classifier (Sensitivity = 0.68, Specificity = 0.75, AUC = 0.81) than for the *Solidago* spp. classifier (Sensitivity = 0.51, Specificity = 0.75, AUC = 0.70), as identified by the internal ten-fold cross-validation procedures. Local thermal conditions (represented by LST) were the most important in the *Erigeron* spp. model (Table 6), while SWIR1 and Aerosols were relevant for *Solidago* spp. occurrence. Four RS variables were relevant in both Asteraceae genera classifiers: RedEdge1, CHMmean, SeLImin and RaoQ_NDVI. The predicted occurrence of both genera separately and the co-invasion maps (Fig. 6) revealed different proportions of these three presence categories across the three ecologically distinct sites (Table 7). In Site1 (with a large proportion of high vegetation) the *Solidago* spp. (77% predicted cover of suitable vegetation patches) dominated over the *Erigeron* spp. (20%); in Site2 (large proportion of bare ground) predicted cover was intermediate for both the IP genera (42% for the *Erigeron* spp., 31% for the *Solidago* spp.); in Site3 (mostly covered by low vegetation) the *Solidago* spp. predicted cover (60%) was considerably higher than the *Erigeron* spp. cover (38%). The proportion of the co-invaded areas was highest in Site3 (33%), then in Site1 (17%) and the smallest in Site2 (13%). The mean pixel values for the most important RS predictors, across the three sites, were always significantly different between the two Asteraceae genera (Table 8), but Cohen’s d revealed that the actual effect sizes were sometimes small (Fig. S2). Relevant to our third objective, the distributions of RedEdge1 were most similar between the two IP genera and were also most consistent across the three sites (RedEdge1 reflectance around 0.1), precisely identifying the co-invaded pixels (Fig. 7; Table 8). In contrast, the mismatch between both genera niches was best captured by LST (averaging between 37.5 and 45.3 °C for the *Erigeron* spp. and between 33.2 and 40.6 °C for the *Solidago* spp.) and by the LiDAR-derived CHMmean (averaging between 0.12 and 0.56 m for the *Erigeron* spp. and between 1.7 and 5.1 m for the *Solidago* spp.). Overall, the habitats suitable for the *Erigeron* spp. were more frequent in the initial successional stage (50% of the bare ground LULC) and the habitats suitable for the *Solidago* spp. dominated in the late successional stage (65% of the high vegetation LULC), both genera attained high potential coverages (well above 50%) in the intermediate stage (Table 9; Fig. 8). The proportions of co-invaded areas in the LULC classes were as follows: low vegetation (58%) > built area (45%) > bare ground (9%) > high vegetation (6%). The site-specific percentages in the two well represented classes (high and low vegetation) agreed well with the overall predictions (Table 9).

**Table 6:**
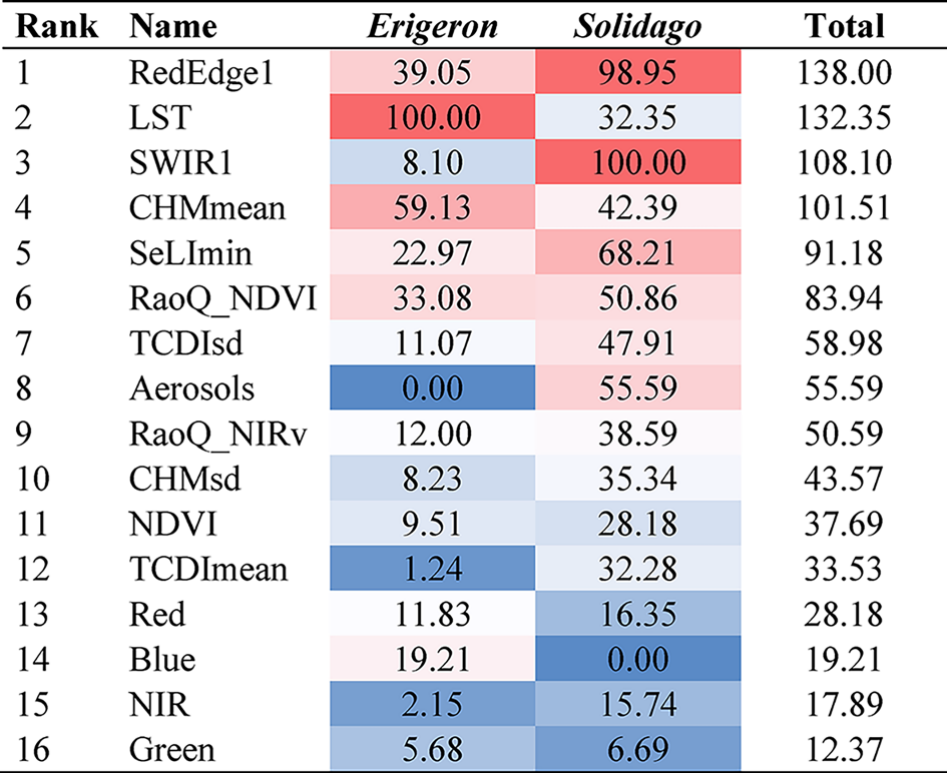
Scaled variable importance, based on the GBM models trained using the full RS dataset (*n* = 358), ranked by the total importance in both *Erigeron* spp. and *Solidago* spp. models; different colors highlight importance in individual models: from highest (red) to lowest (blue).

**Table 7:** Site-level GBM-predicted cover (in hectares and as site area percentage) of *Erigeron* spp., *Solidago* spp. and both genera co-occurrence (Co-invasion).

	*Erigeron* ha (%)	*Solidago* ha (%)	Co-invasion ha (%)
Site1	33.9 (20.2%)	128.6 (76.8%)	28.2 (16.8%)
Site2	47.5 (41.5%)	35.6 (31.2%)	14.3 (12.5%)
Site3	44.6 (37.5%)	71.3 (59.8%)	39.5 (33.2%)

**Table 8:** Summary statistics of site-level remote sensing data pixel values (Site1-3) and subsets identified by the GBM predictions for *Erigeron* spp. occurrence (Erigeron), *Solidago* spp. (Solidago) occurrence or both genera co-occurrence (Co-invasion); Welch test *p*-values denote non-zero differences between Erigeron and Solidago and Cohen’s d indicates both the effect size and direction of the difference (positive values for larger erigeron mean and negative values for larger Solidago mean).

Site1 (*n* = 66,974)							
No.	Name (unit)	Site1 mean (± SD)	Erigeron mean (± SD)	Solidago mean (± SD)	Welch test *p*-value	Cohen’s d	Co-invasion mean (± SD)
1	LST (°C)	33.07 (± 4.19)	37.78 (± 3.09)	33.19 (± 3.65)	< 0.001	1.3	37.28 (± 2.9)
2	SeLImin (-)	0.4 (± 0.21)	0.19 (± 0.14)	0.39 (± 0.19)	< 0.001	-1.17	0.21 (± 0.13)
3	RedEdge1 (-)	0.08 (± 0.03)	0.1 (± 0.02)	0.08 (± 0.02)	< 0.001	0.82	0.1 (± 0.02)
4	CHMmean (m)	6.25 (± 6.75)	0.56 (± 1.4)	5.05 (± 5.61)	< 0.001	-0.89	0.65 (± 1.5)
5	Aerosols (-)	0.03 (± 0.02)	0.04 (± 0.02)	0.03 (± 0.01)	< 0.001	0.75	0.04 (± 0.02)
6	RaoQ_NDVI (-)	0.08 (± 0.07)	0.14 (± 0.07)	0.09 (± 0.07)	< 0.001	0.61	0.14 (± 0.07)

**Site2 (n = 45**,**754)**							
**No.**	**Name (unit)**	**Site2** **mean (± SD)**	**Erigeron** **mean (± SD)**	**Solidago** **mean (± SD)**	**Welch test** *p*-value	**Cohen’s d**	**Co-invasion** **mean (± SD)**
1	LST (°C)	43.27 (± 5.25)	45.34 (± 3.06)	40.6 (± 4.06)	< 0.001	1.35	43.08 (± 3.24)
2	SeLImin (-)	0.18 (± 0.2)	0.1 (± 0.09)	0.26 (± 0.15)	< 0.001	-1.37	0.17 (± 0.1)
3	RedEdge1 (-)	0.06 (± 0.02)	0.07 (± 0.01)	0.07 (± 0.02)	0.033	-0.02	0.08 (± 0.01)
4	CHMmean (m)	2.16 (± 3.82)	0.18 (± 0.41)	2.92 (± 3.76)	< 0.001	-1.1	0.3 (± 0.67)
5	Aerosols (-)	0.03 (± 0.01)	0.04 (± 0.01)	0.03 (± 0.01)	< 0.001	1.01	0.04 (± 0.01)
6	RaoQ_NDVI (-)	0.07 (± 0.06)	0.09 (± 0.06)	0.1 (± 0.06)	< 0.001	-0.27	0.11 (± 0.06)

**Site3 (n = 47**,**646)**							
**No.**	**Name (unit)**	**Site3** **mean (± SD)**	**Erigeron** **mean (± SD)**	**Solidago** **mean (± SD)**	**Welch test** *p*-value	**Cohen’s d**	**Co-invasion** **mean (± SD)**
1	LST (°C)	34.12 (± 4.33)	37.5 (± 3.46)	35.48 (± 3.69)	< 0.001	0.56	37.15 (± 2.96)
2	SeLImin (-)	0.45 (± 0.17)	0.39 (± 0.11)	0.43 (± 0.11)	< 0.001	-0.34	0.4 (± 0.09)
3	RedEdge1 (-)	0.08 (± 0.02)	0.1 (± 0.01)	0.09 (± 0.02)	< 0.001	0.7	0.1 (± 0.01)
4	CHMmean (m)	3.33 (± 4.94)	0.12 (± 0.51)	1.74 (± 3.59)	< 0.001	-0.57	0.12 (± 0.47)
5	Aerosols (-)	0.02 (± 0.01)	0.02 (± 0)	0.02 (± 0)	< 0.001	0.49	0.02 (± 0)
6	RaoQ_NDVI (-)	0.04 (± 0.04)	0.04 (± 0.04)	0.04 (± 0.04)	< 0.001	-0.1	0.04 (± 0.03)

**Table 9:** Combined percentage predicted presence across the three selected sites, for *Erigeron* spp., *Solidago* spp. and both genera co-occurrence (Co-invasion) in four broad land cover classes (reclassified from dynamic world LULC^28^; for the two classes well represented across the three sites (high and low vegetation) site-specific predictions are given in parentheses (from Site1 to Site3, respectively).

Predicted	High vegetation (%)	Low vegetation (%)	Bare ground (%)	Built area (%)
*Erigeron*	7.0 (7.4, 10.7, 3.2)	67.8 (48.7, 61.8, 71.5)	49.8	58.4
*Solidago*	64.6 (77.9, 52.1, 41.3)	81.2 (93.7, 70.0, 81.7)	11.3	70.6
Co-invasion	6.0 (7.0, 7.0, 3.0)	57.5 (45.2, 36.1, 63.5)	8.5	45.4

## Discussion

### **Local thermal conditions drive*Erigeron*spp. distribution**,** but local moisture was more important for*Solidago*spp.**

Our study revealed promising avenues for predicting IP genera co-occurrence on local and regional scales using a combination of fine to moderate resolution RS data (5–60 m), which integrates multispectral and LiDAR information. Following other studies comparing machine learning techniques, the ensemble-based methods performed better than the kernel-based method (SVM), and the flexibility of the stochastic GBM proved most advantageous^71,72^. Our analyses identified that LST (maximal summer surface temperature, 30-m resolution) carried the most relevant information for the *Erigeron* spp. habitat suitability, which agrees very well with their biological and ecological needs^73,74^ as early invaders. Land Surface Temperature was always ranked high in the predictor importance lists, also in the *Solidago* spp. models, but in the latter case there were some more relevant RS variables, particularly the SWIR1 Sentinel-2 (20-m resolution) band. This reflectance band is highly indicative of vegetation and soil water content, due to the extreme absorption by water in this part of the spectrum^75^. Moreover, SWIR1 noticeably increased in importance after removal of the FD (field-based) predictors, which may suggest that relevant habitat information was overlapped by both FD predictors and SWIR1 (further supported by a high Variance Inflation Factor of SWIR1 in the RDA model, cf. Supplementary Table S3). Indeed, these local characteristics agree with the previously identified broad-scale climatic differences between both genera, for instance, the humid climate of the Zhenjiang (China) zone was found to be suboptimal for *Erigeron canadensis*, compared to the warm temperate continental climate of the Jinan zone^76,77^, while the climatic pattern in China was opposite in the case of *Solidago canadensis*^19^.

### Spectral diversity and LiDAR-based vegetation height jointly contribute to explaining community-level invasibility

The major axis of the RDA ordination captured similarities between both Asteraceae genera and represented a community-level invasibility gradient. Our results support the concept of a spectrally-derived Rao’s Q diversity index^54^ being a surrogate of the field-based functional diversity^55^. Moreover, indeed high taxonomic and functional richness as well as the RaoQ_NDVI index were positively related to probability of both IP genera occurrence^7^. In contrast, high Specific Leaf Area, Seed Mass of native plants and community-level plant height (including the RS CHMmean metric) limited habitat invasibility. This supports the findings that both IP genera are important invaders in communities that can be outcompeted by a rapid height growth and tall stature of the IPs^78^. In fact, it appears that plant height of the IPs is more relevant for the overall advantage over native plants than leaf size and photosynthetic capabilities^78^. Moreover, the rhizomatous *Solidago* spp. may inhibit natural succession of taller woody species for decades, thus degrading the potentially much higher ecosystem services^79^.

### Sentinel-2 RedEdge1 band best identified co-invaded areas

We found a limited niche overlap and potential co-invasion area of the two Asteraceae genera in the studied landscapes, in contrast to the commonly reported co-invasion of the genera representatives in warmer regions of Eurasia^6–8,13^. Still, there was a considerable overlap of the suitable habitat patches for both genera, at least in the low vegetation site (Site3), primarily due to limited competition of native plants^2,3^. Accordingly, the low vegetation LULC type was most likely to be co-invaded across the three test sites, with the most equal cover of both genera (slightly dominated by the *Solidago* spp.). Interestingly, the built area LULC type was the second most co-invaded (also dominated by the *Solidago* spp.), primarily due to a high propagule pressure^2,3^. In terms of the RS predictors, this was reflected by a high importance of the local disturbance metrics (such as TCDIsd) in the *Solidago* spp. models. Overall, RedEdge1 was the most relevant RS predictor, identifying potential co-invasion of both Asteraceae genera, in a narrow spectral range across the three sites. This important result supports the findings of another study^80^, which also identified the Sentinel-2 red-edge spectral range as the most relevant for capturing specific properties of vegetation. That study aimed at machine learning modeling of a xeric (semi-arid, Caatinga) ecosystem in northeast Brazil, using a very similar set of RS predictors to our study (Sentinel-2 multispectral and LiDAR height information). These results further confirm that the steep change from chlorophyll absorption to foliage internal structure reflectance in the red-edge interval maximizes the differences between vegetation characteristics^81,82^. We only focused on the two Asteraceae genera, but it is well known that the native expansive grass *Calamagrostis epigejos* dominates in many early successional parts of the landscape^15^. Future studies may aim at elucidating if the red-edge spectral range is also useful for identifying differences within vegetation functional groups.

### High FRic of native plants may limit invasiveness of the two Asteraceae genera

Following our expectation, the plot-level Functional Richness calculated for all plant species (FRic.all) and the version calculated for native plants only (FRic.nat) worked differently in all types of models and also differed between the two Asteraceae genera classifiers. While FRic.all was generally the strongest predictor across this study, increasing the probability of the IP genera presence, the Fric.nat appeared to either counteract invasiveness (as in the GBM and SVM models) or pose no considerable influence (as in the RF models). On the one hand, these results agree with the biotic resistance hypothesis^48,49^ and the theoretical rationale behind a high Functional Richness^50^, which should increase the community resistance to invasions when the functional niche is already filled by native plants. On the other hand, these findings call for more frequent and careful analyses of native versus total plant compositions in similar analyses^7,14^, to avoid drawing overly broad conclusions about the effects of IPs on plant communities. Further studies may directly target these differences, probably by including narrow-band hyperspectral data^46^, while none of our RS predictors seemed to explicitly capture the different effects of FRic.all and FRic.nat. This may be attributed to the well-known inconsistency problem when scaling from plot-level experimental data to landscape level RS data^20^.

## Conclusion

This research proposed a comprehensive framework for machine learning modeling of post-industrial habitats prone to the co-invasion of two Asteraceae invasive plant genera (*Erigeron* spp. and *Solidago* spp.), using moderate to fine-resolution remote sensing data, based on presence-absence records. Stochastic Gradient Boosting (GBM) best captured the often non-linear effects of predictors and generally outcompeted the two other machine learning methods (Random Forest and Support Vector Machines with a Radial Basis Function Kernel). The predictive power of field-based variables (such as Functional Richness of all plant species and the successional gradient) was larger than that of the remote sensing predictors alone. Certain links between the former and the latter datasets were identified using a canonical ordination method. Functional Richness and RS-based spectral diversity indices (such the Rao’s Q entropy) worked similarly in the models, positively influencing habitat invasibility. The community weighted mean functional traits (such as Seed Mass and Specific Leaf Area) were positively related with the LiDAR-derived local vegetation height metrics, and all counteracted invasiveness by the two Asteraceae IP genera. The most favorable conditions for co-invasion, in terms of remotely sensed data, were identified by a narrow range of reflectance in the first red-edge band of a Sentinel-2 image. Importantly, we found that the share of patches suitable for co-invasion was consistently highest in the low vegetation land cover class, between 36% and 64% cover. Presence of these IPs may inhibit natural succession, we therefore advise considering particular management actions, such as increasing the supply of native seed, thus improving local community resistance to invasions. The proposed methods and RS predictors may facilitate targeted monitoring and cost-optimized management interventions.

## Supplementary Information

Below is the link to the electronic supplementary material.

Supplementary Material 1

## Data Availability

The field data are available from a public repository (https://doi.org/10.6084/m9.figshare.25289401). Sentinel-2 data were downloaded and are available from the Copernicus Browser (https://browser.dataspace.copernicus.eu), Landsat 7 data are available via the Google Earth Engine web-based facility, other data are available from the corresponding author upon a reasonable request.
