# Supplementary material for: Predicting the co-invasion of two Asteraceae plant genera in post-mining landscapes using satellite remote sensing and airborne LiDAR
Source: Sci Rep. 2025 Oct 7;15:34877. doi: 10.1038/s41598-025-16441-3 (PMC12504456; doi:10.1038/s41598-025-16441-3)
Supplement: Supplementary file 1 — Supplementary Material 1 [file 41598_2025_16441_MOESM1_ESM.pdf]

Supplementary Information

Title: Predicting the co-invasion of two Asteraceae plant genera in post-mining landscapes using satellite remote sensing and airborne LiDAR

Kamil Kędra*, Andrzej M. Jagodziński

Institute of Dendrology, Polish Academy of Sciences, Kórnik, Poland

*k.w.kedra@gmail.com

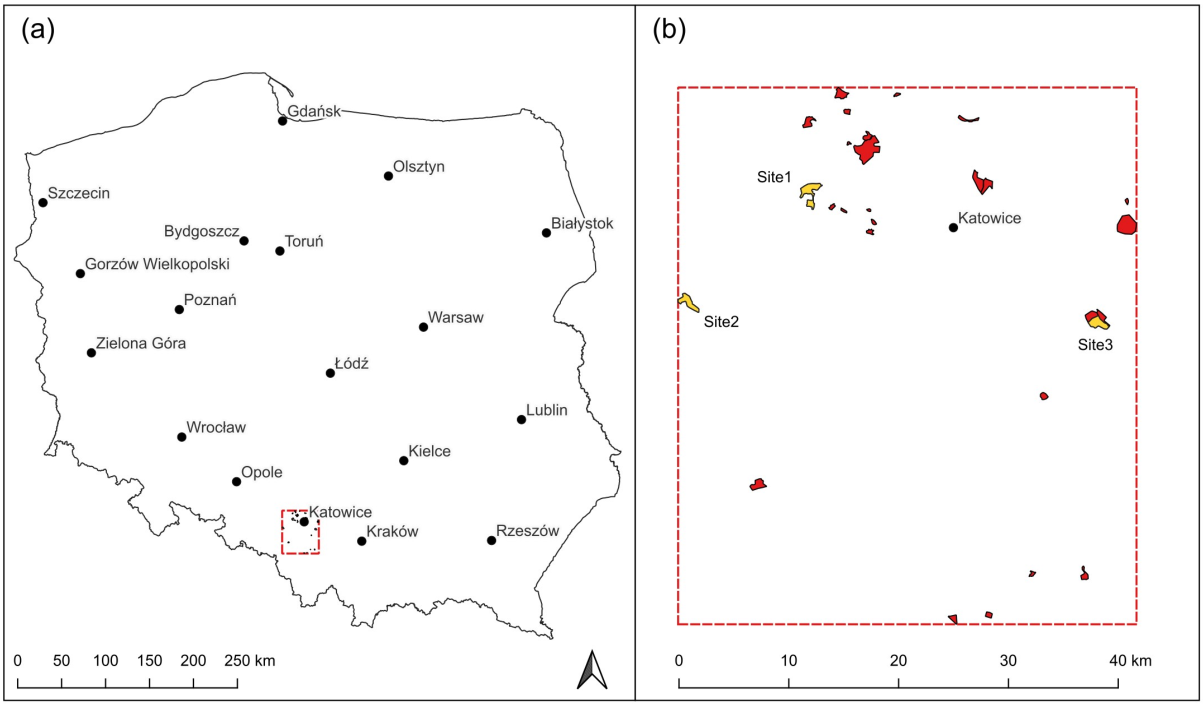

Figure S1. Study area (red dashed rectangle) within the borders of Poland (black outline), black dots indicate locations of major cities (a); close-up of the study area including 28 spoil heaps (red or yellow polygons), the three sites (Site1-3) selected for pixel-based machine learning Asteraceae genera occurrence predictions are highlighted by yellow fill (b).

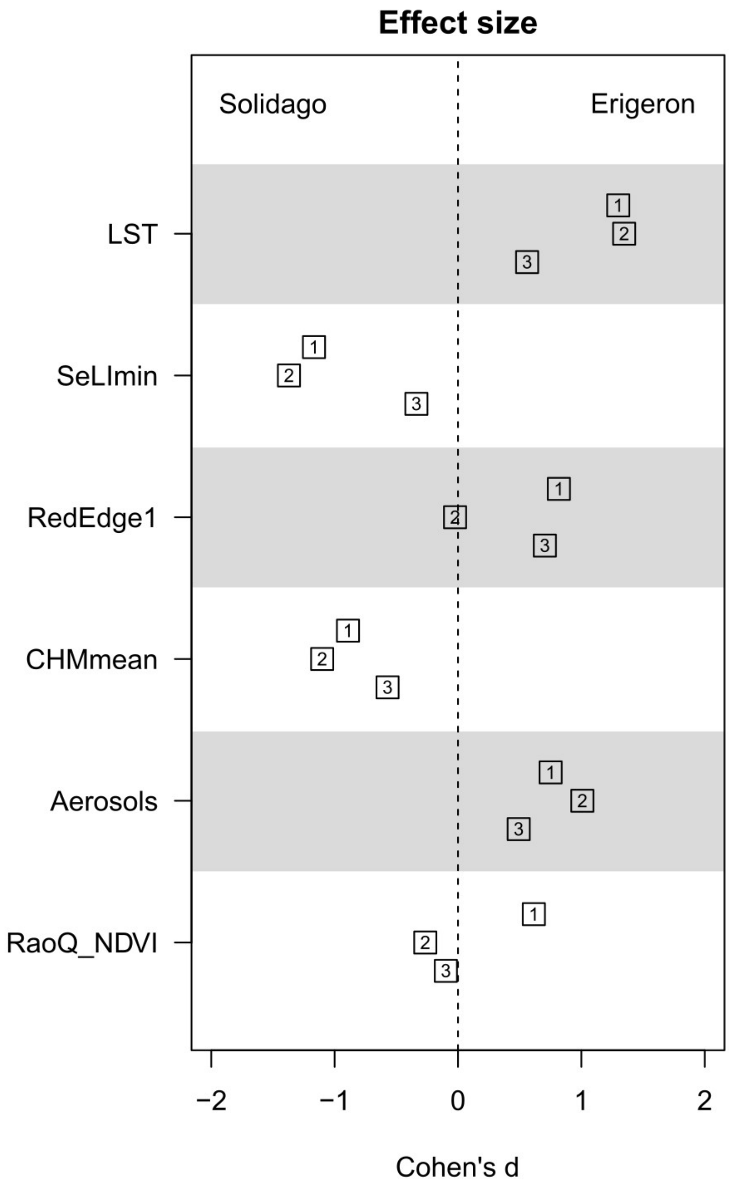

Figure S2. Effect size (Cohen's d) for the most influential RS variables in the final GBM models of *Erigeron* spp. and *Solidago* spp. occurrence; positive values indicate higher mean values within the Erigeron-present pixels and negative values indicate higher mean values within the Solidago-present pixels; different digits (1-3) denote different sites (cf. Fig. 2 in the main text).

No.	Name	GBM_Er	SVM_Er	RF_Er	Total
1	FRic.all	100.00	100.00	100.00	300.00
2	Succession	53.74	74.23	68.93	196.90
3	nbsp.all	17.70	91.87	34.55	144.12
4	LST	7.63	64.66	23.14	95.44
5	FRic.nat	20.64	59.27	8.91	88.83
6	nbsp.nat	0.00	74.71	4.93	79.63
7	TCDImean	11.22	52.22	14.97	78.41
8	CHMmean	9.89	62.06	5.13	77.09
9	Blue	13.36	51.53	6.32	71.20
10	SeLImin	6.27	49.67	12.85	68.79
11	Red	11.64	49.93	5.36	66.93
12	RedEdge1	11.04	42.92	9.06	63.02
13	NIR	10.34	46.19	4.87	61.39
14	RaoQ_NDVI	8.25	49.64	2.51	60.39
15	H.all	7.65	52.05	0.00	59.70
16	H.nat	7.75	47.08	3.56	58.39
17	Green	3.33	46.14	6.62	56.08
18	Aerosols	11.00	41.94	3.01	55.96
19	CHMsd	4.81	50.76	0.20	55.77
20	SM.all	8.16	43.76	2.40	54.31
21	NDVI	1.87	47.79	2.14	51.79
22	SM.nat	4.24	36.12	1.56	41.92
23	SLA.all	3.28	28.00	4.76	36.03
24	SLA.nat	2.52	27.53	5.89	35.95
25	SWIR1	2.86	26.65	5.91	35.42
26	RaoQ_NIRv	4.51	21.52	8.21	34.24
27	TCDIsd	9.00	0.00	4.16	13.16

Table S1. Scaled variable importance for machine learning *Erigeron* spp. presence-absence classification models (GBM, SVM, RF) fitted with fused field and remote sensing data, ordered by the Total importance.

No.	Name	GBM_So	SVM_So	RF_So	Total
1	nbsp.all	100.00	100.00	91.32	291.32
2	FRic.all	63.04	58.29	96.89	218.22
3	Succession	45.06	25.01	100.00	170.06
4	FRic.nat	36.47	60.65	28.66	125.78
5	SM.all	41.75	39.22	42.24	123.22
6	nbsp.nat	5.65	83.77	19.09	108.51
7	SLA.all	26.79	37.38	31.72	95.89
8	Aerosols	30.87	14.86	42.04	87.77
9	SeLImin	18.71	26.23	42.14	87.08
10	NDVI	13.99	29.60	31.88	75.47
11	LST	25.17	18.81	30.22	74.20
12	SWIR1	14.27	18.73	33.09	66.08
13	Red	23.60	20.78	20.17	64.54
14	SLA.nat	19.73	21.87	21.91	63.51
15	RaoQ_NIRv	21.23	41.27	0.00	62.50
16	Blue	0.00	24.33	37.73	62.06
17	CHMsd	21.57	13.83	23.94	59.33
18	RedEdge1	20.57	14.57	21.82	56.95
19	RaoQ_NDVI	31.62	22.67	2.22	56.51
20	H.nat	34.22	17.74	4.33	56.29
21	TCDImean	13.88	9.53	27.08	50.49
22	TCDIsd	32.84	5.27	12.09	50.20
23	CHMmean	19.08	6.74	22.03	47.85
24	H.all	33.75	5.53	7.67	46.95
25	NIR	10.84	27.32	8.46	46.62
26	SM.nat	26.35	0.00	12.96	39.31
27	Green	2.10	9.08	18.65	29.82

Table S2. Scaled variable importance for machine learning *Solidago* spp. presence-absence classification models (GBM, SVM, RF) fitted with fused field and remote sensing data, ordered by the Total importance.

(a)	Name	VIF
	nbsp.nat	37.83
	FRic.nat	15.13
	SLA.nat	4.07
	SM.nat	1.82
	H.nat	15.81
	nbsp.all	45.22
	FRic.all	12.44
	SLA.all	5.21
	SM.all	1.55
	H.all	17.02
	Succession	8.04
	CHMmean	13.71
	CHMsd	9.17
	NDVI	14.26
	LST	21.15
	Aerosols	19.19
	Blue	203.37
	Green	450.23
	Red	513.94
	RedEdge1	67.99
	NIR	32.47
	SWIR1	130.16
	TCDImean	296.11
	TCDIsd	8.37
	SeLImin	63.89
	RaoQ_NDVI	11.38
	RaoQ_NIRv	10.09

(b)	Name	RDA1	RDA2
	nbsp.nat	0.66	-0.12
	FRic.nat	0.74	0.07
	SLA.nat	-0.56	-0.08
	SM.nat	-0.30	-0.06
	H.nat	-0.30	-0.09
	nbsp.all	0.71	0.01
	FRic.all	0.82	0.17
	SLA.all	-0.54	-0.03
	SM.all	-0.01	-0.28
	H.all	-0.31	-0.10
	Succession	-0.49	-0.43
	CHMmean	-0.61	-0.24
	CHMsd	-0.60	-0.23
	NDVI	-0.24	-0.54
	LST	0.42	0.52
	Aerosols	0.23	0.48
	Blue	0.06	0.43
	Green	0.12	0.35
	Red	0.09	0.39
	RedEdge1	0.26	0.26
	NIR	-0.14	-0.47
	SWIR1	0.24	0.12
	TCDImean	0.22	0.40
	TCDIsd	0.18	0.17
	SeLImin	-0.44	-0.49
	RaoQ_NDVI	0.57	-0.09
	RaoQ_NIRv	0.33	-0.50

Table S3. Redundancy Analysis (RDA) statistics: Variance Inflation Factors (VIF), values above 10 indicate redundant constraints (a); RDA scores, coordinates of arrows in the RDA biplot (b); see main text Tab. 2 for explanations of abbreviations and Fig. 4 for the RDA biplot; in both (a) and (b) field data variables (top) are separated from remote sensing variables (bottom) by a black horizontal line.

No.	Name	GBM_Er	SVM_Er	RF_Er	Total
1	LST	100.000	100.000	100.000	300.000
2	SeLImin	39.651	76.816	67.772	184.239
3	RedEdge1	38.581	66.368	74.043	178.992
4	Blue	21.618	79.682	55.990	157.289
5	TCDImean	9.002	80.753	59.611	149.365
6	CHMmean	8.791	95.977	37.627	142.395
7	Red	8.685	77.221	50.063	135.970
8	Green	9.070	71.346	49.202	129.619
9	Aerosols	9.044	64.863	35.854	109.761
10	NDVI	3.778	73.907	29.748	107.434
11	RaoQ_NDVI	12.647	76.758	1.852	91.257
12	CHMsd	0.000	78.495	10.424	88.918
13	NIR	7.458	71.433	0.000	78.890
14	SWIR1	12.276	41.216	2.030	55.522
15	RaoQ_NIRv	9.361	33.285	5.994	48.641
16	TCDIsd	11.688	0.000	18.474	30.163

Table S4. Scaled variable importance for machine learning *Erigeron* spp. presence-absence classification models (GBM, SVM, RF) fitted with remote sensing data, ordered by the Total importance.

No.	Name	GBM_So	SVM_So	RF_So	Total
1	SWIR1	100.000	37.392	49.239	186.631
2	SeLImin	49.394	58.213	77.203	184.810
3	Aerosols	44.075	26.657	100.000	170.732
4	RedEdge1	51.244	25.829	47.171	124.243
5	RaoQ_NIRv	11.947	100.000	4.343	116.290
6	NDVI	14.317	67.579	33.369	115.265
7	Blue	22.928	52.954	31.626	107.508
8	TCDIsd	56.740	0.000	34.136	90.876
9	RaoQ_NDVI	41.570	48.343	0.000	89.913
10	NIR	13.462	61.239	11.616	86.318
11	LST	13.853	37.608	33.319	84.780
12	Red	4.529	43.084	11.487	59.099
13	CHMsd	29.565	23.775	4.988	58.328
14	TCDImean	15.122	11.852	24.394	51.367
15	Green	7.391	10.591	14.610	32.592
16	CHMmean	0.000	4.107	12.761	16.868

Table S5. Scaled variable importance for machine learning *Solidago* spp. presence-absence classification models (GBM, SVM, RF) fitted with remote sensing data, ordered by the Total importance.

Method	Acronym	Tuned Parameters FD+RS Erigeron	Tuned Parameters FD+RS Solidago	Tuned Parameters RS Erigeron	Tuned Parameters RS Solidago
Stochastic Gradient Boosting	GBM	n.trees=150, interaction.depth=5, shrinkage=0.1, n.minobsinnode=20	n.trees=700, interaction.depth=5, shrinkage=0.1, n.minobsinnode=20	n.trees=100, interaction.depth=1, shrinkage=0.1, n.minobsinnode=20	n.trees=100, interaction.depth=1, shrinkage=0.1, n.minobsinnode=21
Support Vector Machines with Radial Basis Function Kernel	SVM	sigma=0.04879442, C=4	sigma=0.04879442, C=32	sigma=0.1243359, C=0.25	sigma=0.1243359, C=1
Random Forest	RF	mtry=10	mtry=2	mtry=2	mtry=2

Table S6. Tuned hyperparameters for the three modeling methods (GBM, SVM, RF) across the two modeling parts (dataset configurations): field plus remote sensing data (FD+RS) and RS data only, for both *Erigeron* spp. and *Solidago* spp.
